# Nanomaterials in COPD: Emerging Therapeutic and Diagnostic Frontiers with a Focus on Metal–Organic Frameworks

**DOI:** 10.3390/ijms26168025

**Published:** 2025-08-19

**Authors:** Antonio Tiralosi, Manuela Cambria, Mariachiara Campanella, Vincenzo Paratore, Cristina Russo, Lucia Malaguarnera, Maria Stella Valle, Maria Teresa Cambria

**Affiliations:** 1 Volunteers Organization, Via re Martino 84a, 95126 Catania, Italy; tiralosiantonio@gmail.com (A.T.); manu.cambria@icloud.com (M.C.); 2 Section of Medical Biochemistry, Department of Biomedical and Biotechnological Sciences, University of Catania, 95123 Catania, Italy; mariachiara.campanella97@gmail.com; 3 Department of Chemical, Biological, Pharmaceutical and Environmental Sciences, University of Messina, Viale F. Stagno d’Alcontres 31, 98166 Messina, Italy; viparatore@unime.it; 4 Section of Pathology, Department of Biomedical and Biotechnological Sciences, University of Catania, 95123 Catania, Italy; cristina.russo@unict.it (C.R.); lucmal@unict.it (L.M.); 5 Section of Physiology, Department of Biomedical and Biotechnological Sciences, University of Catania, 95123 Catania, Italy; m.valle@unict.it

**Keywords:** chronic obstructive pulmonary disease, nanomaterials, MOFs, inhalable drug delivery, alveolar targeting

## Abstract

Chronic obstructive pulmonary disease (COPD) is one of the leading causes of morbidity and mortality worldwide. Although conventional therapies are effective in controlling symptoms, they remain limited in altering the course of the disease and significantly reducing the chronic inflammation and oxidative stress underlying it. In this context, nanoparticles and nanomaterials are emerging as innovative tools capable of overcoming traditional pharmacological barriers due to their ability to deliver therapeutic oligonucleotides, antioxidants, and drugs in a targeted manner, modulate immune responses, and improve the bioavailability of active compounds. In particular, metal–organic frameworks (MOFs) stand out as ideal candidates for inhalable drug delivery in COPD, owing to their permanent crystalline porous structure, high specific surface area, and versatile chemical functionalization. This review provides the most recent preclinical evidence on the use of different nanoparticles in COPD, with a focus on the therapeutic and diagnostic potential of MOFs. It discusses their biocompatibility, drug loading strategies, and controlled release mechanisms and explores future perspectives for clinical translation.

## 1. Introduction

Chronic obstructive pulmonary disease (COPD), the third leading cause of death worldwide, is defined as a pathological condition characterized by persistent respiratory symptoms and airflow limitation that is not fully reversible. The disease encompasses several conditions including emphysema, marked by alveolar destruction, chronic bronchitis, and small airway disease, in which the small bronchioles are reduced in both number and diameter [1]. The primary etiological factors include long-term exposure to tobacco smoke, pollutants, and biomass, which induce a chronic inflammatory response in the airways, structural damage, and remodeling of lung tissue.

Although the clinical manifestations and progression rates of COPD can vary widely among individuals, most patients with stable disease exhibit reduced peripheral muscle endurance and pronounced fatigability [2]. This condition significantly limits physical activity, creating a vicious cycle that exacerbates muscular dysfunction and progressively impairs exercise capacity. Biochemical and histological studies have documented key changes in skeletal muscle among COPD patients, including disrupted enzymatic activity and mitochondrial abnormalities [3], a reduction in muscle fiber cross-sectional area [4], a reduction in muscular stiffness and viscosity [5], and a fiber-type shift from slow-oxidative to fast-glycolytic phenotypes [6]. These alterations contribute to increased muscle fatigue, weakness, and eventual atrophy, particularly in the lower limbs.

Recent studies have highlighted the therapeutic potential of combining aerobic exercise with nano-selenium supplementation, an antioxidant and anti-inflammatory agent, in experimental COPD models [7]. This approach has been associated with elevated levels of beneficial myokines such as irisin and semaphorin 3A (Sema3A) in both serum and lung tissue. These findings suggest that myokines may exert paracrine and endocrine effects, modulating the function and regeneration of distant organs like the lungs [7]. In COPD, the persistent inflammatory state disrupts the oxidant–antioxidant balance. Excessive production of reactive oxygen species (ROS), coupled with impaired clearance mechanisms, leads to oxidative stress and cellular damage. This condition, further aggravated by cigarette smoke and environmental pollutants, activates NF-κB pathways and promotes the release of pro-inflammatory cytokines (e.g., IL-6, TNF-β, IL-1β). The resulting cascade promotes epithelial damage, tissue remodeling, and mitochondrial dysfunction. Additionally, impaired mitophagy and elevated mitochondrial ROS further contribute to disease progression [8,9,10,11]. Current pharmacological treatments primarily aim to alleviate symptoms and reduce exacerbations, but they do not target the underlying disease mechanisms, underscoring the need for more effective and targeted therapeutic strategies. In this context, the unique anatomical and physiological features of the lung, particularly its vast surface area, make it an attractive target for inhalation-based drug delivery. Nanoparticles have emerged as promising tools in both the diagnosis and treatment of lung diseases. However, their application in pathological conditions such as COPD must be approached cautiously, as certain nanoparticles can trigger cytotoxicity and stimulate pro-inflammatory cytokines like IL-1β, IL-6, and IL-18. Conversely, some nanomaterials exhibit antioxidant properties, either by neutralizing free radicals directly or by modulating mitochondrial ROS release, thereby potentially mitigating oxidative damage in lung cells.

The advent of nanotechnology has thus opened new frontiers in medicine. Nanoparticles, due to their nanoscale dimensions and customizable surface chemistry, offer precise and controlled drug delivery, reducing systemic side effects and enhancing therapeutic outcomes. Particle size directly influences deposition within the respiratory tract, with optimal aerodynamic diameters (~1–5 µm) required for deep lung penetration. Formulation stability, particularly in liposomal carriers, is equally crucial to ensure consistent dosing, maintain encapsulated drug integrity, and prevent premature leakage or aggregation. Together, these factors substantially impact the bioavailability, safety, and clinical viability of inhaled therapeutic formulations, especially in chronic respiratory conditions such as COPD. Among these, metal–organic frameworks (MOFs) have garnered particular attention. These porous, crystalline materials are composed of metal ions coordinated with organic ligands, resulting in structures with high surface area, tunable porosity, and functional versatility. In biomedical applications, MOFs have shown promise as drug carriers, biosensors, and imaging agents. Their properties make them especially suitable for pulmonary delivery, where they can respond to specific physiological conditions, encapsulate therapeutic agents, and release them in a controlled fashion. MOFs are currently being explored not only for targeted drug delivery in inflamed or damaged lung tissues in COPD but also for diagnostic purposes, including disease imaging and biomarker detection.

This review aims to provide a comprehensive overview of the role of nanoparticles, with a focus on MOF-based nanomaterials, in the diagnosis and treatment of COPD. Nanoparticles enable controlled and sustained drug release directly at inflamed sites, minimizing systemic exposure and adverse effects, and improving therapeutic outcomes. We discuss their design principles, functional advantages, and translational potential, highlighting their relevance in the emerging field of precision respiratory medicine. By analyzing the latest advances and most promising experimental findings, this work seeks to define the current state of the art and future perspectives for nanotechnology-based interventions in COPD management.

In the subsequent sections, the main types of nanoparticles currently under preclinical investigation for the treatment of COPD will be analyzed, with particular focus on materials, mechanisms of action, and the most relevant experimental results.

## 2. Biodegradable Polymers (PLGA, Chitosan)

### 2.1. PLGA

Among the most widely used biodegradable polymers for inhalation drug delivery are poly (lactic-co-glycolic acid) (PLGA) and chitosan. PLGA is a biodegradable polymer composed of glycolic acid and lactic acid linked by ester bonds. It is FDA-approved due to its excellent biocompatibility and biodegradability, as it is metabolized by the body’s clearance systems into nontoxic substances such as water and CO_2_. Typically, PLGA nanoparticles (NPs) used in pulmonary therapy not only have a small steric footprint, being smaller than 100 nm, but also display elongated or honeycomb-shaped morphologies. These structural features enhance mucosal penetration within the lungs due to the increased surface-to-volume ratio relative to spherical particles.

PLGA has been extensively studied as a delivery system in COPD due to its ability to control drug release over time, protect the encapsulated drug from degradation, and modulate particle size and surface charge [12]. Wu et al. [13] showed that hydrophilic hyaluronic acid-coated PLGA nanoparticles (HA@PLGA) containing polymyxin B, an antibiotic commonly used to treat severe infections caused by resistant Gram-negative bacteria, which are prevalent in patients with acute exacerbations of COPD, exhibited strong interactions with pulmonary mucus. These interactions were due to the nanoparticles’ electrical properties, size, and hydrophilicity, which, in vitro, facilitated deeper lung penetration of the drug compared to the administration of free polymyxin B.

Another study, conducted by Beck-Broichsitter et al. [14], focused on the development of biodegradable nanoparticles encapsulating salbutamol, a β_2_-agonist bronchodilator commonly used to relieve bronchospasm in respiratory conditions such as asthma and COPD. The researchers demonstrated that inhalation of salbutamol encapsulated in polymeric nanoparticles composed of PLGA and poly (vinyl sulfonate-co-vinyl alcohol) enabled controlled drug release, representing a potentially more effective strategy for the treatment of respiratory diseases. Specifically, in an isolated rabbit lung model, prolonged release of salbutamol was observed—the amount recovered in the perfusate was significantly lower compared to that from PLGA-only nanoparticles and the free drug solution, indicating enhanced pulmonary retention and more effective delivery of the active compound [14].

Another innovative lipid–polymer nanoparticle (LPN) system was developed with a core–shell structure, in which the PLGA core encapsulates a potent antioxidant, Mn-porphyrin dimer (MnPD), while the outer cationic lipid shell made of DOTAP carries the pHDAC2 plasmid (a genetic construct used to express HDAC2—Histone Deacetylase 2—in eukaryotic cells). The combination of pHDAC2 transfection with the antioxidant action of MnPD led to a marked increase in intracellular HDAC2 levels (a protein largely involved in the repression of pro-inflammatory gene transcription), indicating that the multiple-antioxidant capacity of MnPD is crucial for stimulating its expression [15]. Due to the significant inflammatory impact in the pathogenesis and progression of COPD, several studies have employed PLGA-NPs conjugated with anti-inflammatory drugs. Vij et al. [16] developed a therapeutic release system based on PLGA nanoparticle immunoconjugates (PINPs), specifically composed of PLGA–polyethylene glycol (PLGA-PEG-NP) nanoparticles loaded with ibuprofen. This work was performed in murine models, demonstrating a marked reduction in cigarette smoke-induced neutrophilic inflammation. Furthermore, as shown in the work by Saghir et al. [17], nanoparticles composed of thymoquinone (TQ) incorporated into a PLGA–polyvinyl alcohol (PLGA-PVA) delivery system showed a protective effect against bleomycin-induced pulmonary fibrosis—a chemotherapeutic agent known for its pulmonary toxicity as a side effect. This beneficial effect was attributed to the nanoparticles’ ability to reduce lung inflammation and counteract oxidative stress associated with the treatment. In rats treated with both bleomycin and TQ–PLGA–PVA nanoparticles, IL-10 levels were significantly decreased, while TGF-β1 levels, which were markedly increased in rats treated with bleomycin alone, were reduced in those also receiving nanoparticles. The study in [18] showed promising anti-inflammatory therapeutic efficacy of oridonin loaded into PLGA lipid–polymer microparticles (LPMs) in diseases such as COPD and asthma.

PLGA, due to its biological properties, has been employed in delivery systems based on MOFs. The metal–organic framework (MOF) Fe-MIL-101-NH_2_ encapsulating isoniazid was combined with PLGA and leucine via spray drying to create a microparticulate compound with good aerodynamic properties, controlled release, low toxicity, and promising diagnostic–therapeutic potential [19].

In conclusion, drug-loaded nanocomposite microparticles (NCMPs) represent an innovative strategy to improve cellular uptake, extend drug exposition time in lung tissues, and ensure controlled release. One promising approach involves the use of swellable microparticles to transport nanoparticles to the alveoli, where they are typically removed by macrophages. A PLGA nanoparticle system loaded with curcumin and coated with chitosan or chitosan–PEG was obtained via spray drying. The resulting compound consisted of a respirable hydrogel microparticle characterized by high loading capacity (up to 97%), prolonged release, controlled biodegradation, and a reduced inflammatory response as evidenced by the low release of TNF-alpha from inflammatory cells. Moreover, the slow uptake by macrophages suggests a prolonged exposition time in the lungs. Although research on the use of these microparticles for antioxidant delivery is still in its early stages, they show great potential. A critical requirement for the effectiveness of these nanocomposite microparticles (NCMPs) is the spontaneous release of intact nanoparticles following pulmonary deposition. Therefore, further investigations into both in vitro and in vivo release mechanisms will be essential [20]. The main PLGA-based nanoparticles for COPD applications are summarized in Table 1.

### 2.2. Chitosan

Chitosan, derived from the deacetylation of chitin, is another natural polymer extensively studied for respiratory applications. Its ability to interact with epithelial mucosa, enhance cellular penetration, and stimulate the immune response makes it a promising carrier for anti-inflammatory and antioxidant drugs in COPD. To extend the duration of drug action at the pulmonary level in COPD treatment, mucoadhesive polymers have been investigated due to their ability to bind firmly to mucosal surfaces. These materials exhibit structures and functional groups with high affinity for mucus. In particular, the amino groups present in chitosan form electrostatic interactions with the anionic groups of mucus, significantly improving the adhesive capacity of the microparticles. Mucoadhesive solid lipid microparticles (SLMs) based on alginate and chitosan have been developed to enable targeted and efficient delivery of fluticasone propionate (FP), a corticosteroid with potent anti-inflammatory activity commonly employed in the treatment of asthma and chronic obstructive pulmonary disease (COPD). Due to an average aerodynamic diameter (MMAD) ranging from 3.5 to 4.0 μm, these microparticles are good to reach the secondary bronchi, thereby enhancing the localized therapeutic effect of the drug [21]. Polyelectrolyte complexes based on dextran sulfate and chitosan have been developed to produce nebulizable nanodispersions of budesonide. The electrostatic interactions between the two polymers enhance drug loading capacity and effectively prevent burst release. Increasing the concentration of chitosan further improves drug encapsulation within the nanoparticles, which possess a hydrophilic coating that enhances the aqueous solubility of budesonide. Moreover, chitosan facilitates absorption through the respiratory mucosa due to its mucoadhesive properties and its ability to form interactions with mucus, while also acting as an absorption enhancer in the pulmonary environment. At physiological pH (7.4), a controlled release of 96.26% of the drug was achieved within six hours, attributed to polymer degradation and diffusion mechanisms, thereby enabling direct drug exposure to target organs. Overall, biodegradable polymers represent one of the most advanced and promising platforms in respiratory nanomedicine, offering precise control over drug release and favorable interactions with the pulmonary environment, particularly beneficial for the chronic management of COPD [22].

## 3. Liposomes and Nanoemulsions

### 3.1. Liposomes

Liposomes represent one of the most used nanocarriers, thanks to their similarity to cell membranes. This conformation allows them to incorporate both hydrophilic and lipophilic drugs, offering considerable therapeutic flexibility. The use of liposomes in COPD has shown promising results in the delivery of corticosteroids and antioxidants. Previous studies conducted on murine models have shown that liposomes loaded with budesonide or N-acetylcysteine improve pulmonary accumulation and significantly reduce bronchial inflammation compared to free drug administration [23].

Liposomes are particularly studied to overcome the limitations related to the systemic administration of inhaled corticosteroids (ICSs), and for this reason, innovative delivery systems have been developed to transport glucocorticoids directly to the lungs, reducing systemic exposure and associated side effects, allowing precise drug loading and targeted distribution. Weekly administration of budesonide encapsulated in sterically stabilized liposomes could provide therapeutic outcomes comparable to those of conventional daily budesonide therapy in mitigating allergic inflammation [24]. Salbutamol-loaded liposomes, adding a bronchodilator drug belonging to the class of short-acting beta-2 agonists (SABAs) used for the treatment of asthma and COPD, improve therapeutic efficacy by increasing pulmonary retention time and drug concentration at the site of action [25]. Liposomal systems also represent a promising strategy for passive targeting to the lungs, thanks to their ability of internalization following airway deposition. This process is facilitated by the presence of dipalmitoylphosphatidylcholine (DPPC) in the liposomal membrane, a key phospholipid component of endogenous pulmonary surfactant. The structural similarity between liposomal DPPC and that naturally present in the lungs enhances biocompatibility, promoting uptake and absorption by pulmonary cells [26,27]. Inhaled liposomes containing beclomethasone–dipalmitoylphosphatidylcholine (Bec–DPPC) have been shown to preferentially accumulate in the lungs, exhibiting reduced clearance and sustained drug release, features particularly advantageous for the management of chronic respiratory conditions such as COPD [28].

Previous studies have also investigated the use of liposomes as carriers to deliver lipophilic antioxidant compounds such as α-tocopherol and hydrophilic ones such as glutathione, N-acetylcysteine (NAC), and the enzymes SOD and CAT, whose efficacy is often limited by unfavorable physicochemical properties [29]. Acute damage induced by 2-chloroethyl ethyl sulfide (CEES) (a less toxic surrogate of mustard gas, used to study the effects of oxidative stress) was attenuated in a murine model through airway instillation of NAC delivered via liposomes. This treatment resulted in a 59% reduction in the pulmonary permeability index and normalization of pro-inflammatory mediators, including CINC-1, IL-1β, and TNF-α [30]. Liposomes containing *α*-tocopherol, *γ*-tocopherol, *δ*-tocopherol, and NAC were more effective than liposomes containing only NAC or GSH in blocking the CEES-induced inflammatory response and lung injuries [31]. Moreover, liposomes loaded with curcumin and coated with chitosan or hyaluronan improved protection against oxidative damage and stimulated cell proliferation thanks to a synergistic effect between curcumin and hyaluronan [32].

A further advantage of liposomes lies in the possibility of modifying their surface with specific ligands, antibodies, or polymers such as PEG (polyethylene glycol), in order to prolong systemic circulation and selective targeting of receptors expressed in inflamed areas of the lung. This reduces recognition and uptake by macrophages of the mononuclear phagocyte system. Such strategies facilitate active targeting, which is essential for enhancing therapeutic efficacy while minimizing adverse effects.

### 3.2. Nanoemulsions

Nanoemulsions are colloidal systems consisting of an oil phase and an aqueous phase, stabilized by surfactants. They represent a promising technology in the field of drug delivery due to the numerous advantages they offer. They can be administered via different routes, making them extremely versatile, and are capable of encapsulating both hydrophilic and hydrophobic drugs, improving their solubility and absorption. Additionally, they facilitate skin penetration and are suitable for applications in both human and veterinary medicine. However, some limitations are associated with nanoemulsions: the requirement for relatively high concentrations of surfactants and cosurfactants, as well as susceptibility to stability issues caused by variations in temperature and humidity. Moreover, there exists a risk of instability due to Ostwald ripening [33].

A nanoemulsion based on poloxamer 407 was designed to deliver agarwood essential oil in a cigarette smoke-induced cellular model of COPD. Agarwood–NE showed anti-inflammatory properties by reducing the activity of pro-inflammatory cytokines such as IL-8, GDF-15, IL-1β, and IL-1α while stimulating the production of protective cytokines such as GH, IL-10, and Vitamin D-Binding Protein (VDBP). Moreover, agarwood–NE activated cellular defense mechanisms and tissue repair against smoke-induced damage. These results suggest that agarwood nanoemulsion could represent a promising therapeutic strategy for chronic inflammatory diseases such as COPD [34].

A nanoemulsion based on polysorbate 80 was created to deliver budesonide directly into lung tissues [35]. A lower median mass aerodynamic diameter and a higher fine particle fraction (FPF) were observed compared to the commercially available formulation, indicating a potential improvement in therapeutic efficacy for respiratory diseases. The increase in FPF is significant as it indicates a greater proportion of particles with a diameter below 5 μm, ideal for deposition in the deep lung airways. This results in more efficient pulmonary deposition, greater local bioavailability, and fewer systemic side effects, making nanoemulsion a promising strategy for the inhaled delivery of corticosteroids in the treatment of COPD.

Both liposomes and nanoemulsions represent innovative platforms for enhancing the efficacy of inhalation therapies in COPD, particularly with regard to targeted drug release and minimization of systemic adverse effects. Nevertheless, several challenges remain to be addressed, including overcoming the mucus barrier, evading pulmonary clearance mechanisms, and optimizing aerodynamic properties. Considering the aforementioned preclinical findings, these delivery systems warrant further investigation and advancement into later stages of clinical development for the treatment of COPD. The main liposomal and nanoemulsion-based formulations for COPD applications are summarized in Table 2.

### 3.3. Lipid–Polymer Hybrid Nanoparticles (LPNs)

Lipid–polymer hybrid nanoparticles (LPNs) are emerging as advanced drug delivery systems for chronic obstructive pulmonary disease (COPD) due to their ability to combine the stability of polymeric cores with the biocompatibility of lipid shells. LPNs complexed with PLGA and modified with lipidoids, materials containing multiple amine groups and composed of a tetraalkylated amine backbone with different analogs depending on the degree of alkylation, have proven to be more effective in interacting with anionic siRNA than traditional LPNs modified with the commonly used cationic lipid dioleyltrimethylammonium propane (DOTAP) [36,37].

Core–shell LPNs containing an antioxidant Mn-porphyrin dimer (MnPD) and an HDAC2 plasmid increased intracellular HDAC2 levels, suggesting a combined therapeutic effect as both an antioxidant and epigenetic regulator [15].

A novel lipid–polymer hybrid nanoparticle (LPN) has been developed for the targeted delivery of roflumilast to pulmonary macrophages in COPD [38]. Roflumilast is an anti-inflammatory drug belonging to the class of selective phosphodiesterase-4 (PDE4) inhibitors, approved for the treatment of severe chronic bronchitis associated with COPD in patients with a history of frequent exacerbations. A hybrid core–shell system (Man-LPHFNPs@Roflumilast) was developed by combining fluorescent polymeric nanoparticles loaded with roflumilast and lipid vesicles functionalized with mannose. This system was characterized by good colloidal stability, controlled drug release, and mucus-penetrating ability due to its PEG-rich surface. It exhibited high cellular compatibility and was effectively internalized by macrophages through mannose-mediated targeting. For inhalation use, this system was further converted into microparticles via spray drying using polyvinyl alcohol and leucine as excipients. Although scalable production methods already exist, further efforts are focused on optimizing the synthesis and large-scale manufacturing of LPNs. They are considered among the most suitable delivery systems for addressing oxidative stress in chronic obstructive pulmonary disease (COPD).

## 4. Dendrimers

Dendrimers are synthetic, globular, highly branched, and symmetric macromolecules ranging in size from 10 to 100 nm. They have a central core and surface functional groups that can be tailored for specific therapeutic applications. Thanks to their well-defined three-dimensional structure, highly engineerable surface, and ability to incorporate hydrophobic or hydrophilic molecules either within the internal cavities or on the surface, dendrimers are extremely versatile carriers in nanomedicine.

Dendrimer-based formulations, including polyamidoamine (PAMAM), poly-L-lysine (PLL), and PEGylated derivates, offer different advantages, including improved solubility of hydrophobic drugs, sustained drug release, and enhanced biocompatibility. In COPD, they have been studied for the delivery of antioxidants and anti-inflammatory molecules, with promising results in terms of loading efficiency, stability, and controlled release. Nasr et al. [39] developed PAMAM dendrimers loaded with beclomethasone dipropionate, an inhaled corticosteroid (ICS) commonly used in COPD, for nebulization. The study demonstrated increased solubility of the hydrophobic drug, sustained release, and high aerosol performance (as fine particle fraction), confirming the effectiveness of dendrimers as innovative systems for inhalation delivery. Dendrimers are also being studied as oligonucleotide carriers, particularly to ensure their stability and enhance their delivery [40].

In summary, dendrimers, particularly PAMAM and PEGylated forms, show high potential as pulmonary nanocarriers for COPD, though their clinical application requires further studies on pharmacokinetics, safety, and long-term tolerability.

## 5. Metallic Nanoparticles

Metallic nanoparticles and metal oxide nanoparticles represent a rapidly expanding field in biomedical research due to their extraordinary physicochemical properties, including high surface area, catalytic reactivity, and the ability to generate or scavenge reactive oxygen species (ROS). Their synthesis is achieved through the use of reducing agents or oxidizing/precipitating agents. These nanoparticles are inorganic delivery systems used to transport both small pharmacological molecules and macromolecules such as DNA or proteins, improving their stability in biological fluids, as their surface can be coated and functionalized with specific ligands capable of recognizing cell receptors [41]. Some physicochemical parameters of the metal-based nanoparticles are important due to their behavior in the respiratory tract. Important aspects such as aerodynamic characteristics, interaction with the mucociliary clearance system, and efficiency of lung deposition significantly influence the therapeutic efficacy and safety of inhaled nanoparticle-based formulations. Critical factors including particle size distribution, shape, surface charge, density, and hydrophilicity/hydrophobicity determine how these nanoparticles are transported, deposited, and cleared within the airways. These properties are essential for optimizing nanoparticle design for targeted pulmonary drug delivery, especially in the treatment of chronic respiratory diseases like COPD.

Among the most extensively studied metals in the respiratory field are gold (Au), silver (Ag), platinum (Pt), and cerium (Ce), often employed in the form of oxides or as core materials functionalized with specific ligands. Geiser et al. [42] developed inhalable AuNPs (~21 nm) delivered via aerosol in Scnn1b transgenic mice, a model for chronic bronchitis and emphysema (COPD-like features). AuNPs preferentially accumulate in alveolar epithelial cells rather than alveolar macrophages in diseased mice, suggesting direct delivery to injured lung tissue and potential for targeted therapeutic delivery in COPD contexts [42]. In ovalbumin (OVA)-challenged mice (asthma model, used as proxy for chronic pulmonary inflammatory disease), inhaled ~6 nm AgNPs significantly reduced airway inflammation, Th2 cytokine levels, inflammatory cell infiltration, and oxidative stress indicators such as ROS in bronchoalveolar lavage fluid (BALF) [43].

Cerium oxide nanoparticles offer a more advanced and mechanistically promising strategy for inflammatory lung therapies. Nanoceria’s therapeutic potential, through enzymatic ROS scavenging, miRNA delivery (e.g., miR 146a) [44], and NIR-triggered nanozymes (Ce@P) [45], is well demonstrated in acute lung injury and pneumonia models. Nevertheless, concerns about lung-specific oxidative damage at higher doses, and a lack of COPD-specific studies, highlight the need for cautious optimization in delivery, dosing, and chronic disease modeling.

Conversely, a recent study demonstrated that CuO (Copper (II) Oxide) nanoparticles significantly worsen COPD-like damage in vivo by activating the TXNIP–NLRP3 inflammasome pathway, leading to enhanced oxidative stress and pulmonary inflammation. This suggests that CuONPs may aggravate COPD rather than provide therapeutic benefit [46].

In the context of COPD, metallic nanoparticles have shown particular efficacy in counteracting oxidative stress and mitochondrial dysfunction, two key processes in the progression of the disease. Although some evidence supports the use of such metallic nanoparticles in COPD, their application remains controversial due to conflicting experimental data.

## 6. Dextran-Based Systems

Dextran is a natural polysaccharide composed of glucose units, known for its high biocompatibility, low toxicity, and ease of chemical modification. These characteristics make it an ideal candidate for the design of nanoparticles for inhalation drug delivery in COPD. Dextran-based nanoparticles can be engineered to carry anti-inflammatory molecules, antioxidants, or nucleic acids, protecting them from enzymatic degradation and improving their pulmonary bioavailability.

In particular, in a recent study, budesonide was chemically conjugated to dextran via ester bonds, using spacers such as succinic, glutaric, and adipic anhydrides. These conjugates allow for a prolonged release of the drug due to enzymatic degradation by esterases present in the lungs, particularly carboxylesterase 1 [47]. Particles, obtained through spray drying, effectively reach the deep pulmonary regions, prolong the therapeutic effect, and reduce the frequency of administration, an essential aspect to improve treatment adherence in elderly patients.

Recent studies have shown that dextran nanoparticles, especially those crosslinked with functional agents such as tannic acid or polyethylene glycol derivatives, can increase retention time in the respiratory tract and modulate drug release in response to the pH of the inflamed environment [48].

The use of acetylated dextran, obtained by reaction with 2-methoxypropene, has been widely described in the literature and developed for the production of acid-sensitive inhalable microparticles.

These particles, degradable in acidic environments such as lysosomes, enable targeted release of anti-inflammatory drugs such as curcumin and budesonide [49,50]. Their optimal size and negative surface charge favor mucus penetration and resistance to phagocytosis. In particular, the co-encapsulation of budesonide and polydopamine allows for a dual anti-inflammatory and ROS-scavenging effect, inducing macrophage polarization toward the M2 phenotype, which is relevant for the modulation of inflammation in COPD [50]. It should also be noted that dextran can be easily functionalized with targeting peptides or cationic groups, promoting interaction with bronchial epithelial cells and intracellular uptake.

Another promising application involves the use of dextran in the design and development of nanogels. Nanogels are nanoparticles formed by a crosslinked three-dimensional polymeric network that, upon contact with a solvent, rapidly swells, acquiring a gelatinous consistency. These structures combine the features of hydrogels and nanoparticles, due to chemical crosslinking and physical interactions that maintain their integrity, while enabling the absorption of large volumes of water. Their porous network facilitates solute diffusion and enables the encapsulation of different types of drugs, both hydrophilic and hydrophobic. Unlike many rigid nanoparticles, nanogels, once swollen, behave like soft and deformable materials capable of overcoming biological barriers with minimal nonspecific interactions.

Dextran nanogels loaded with dexamethasone were employed for the treatment of pulmonary inflammation, targeting the ICAM-1 protein, an important cellular adhesion molecule [51].

In addition, due to the possibility of incorporating fluorescent or radio-opaque diagnostic agents, dextran-based systems lend themselves to advanced theranostic therapies. Specifically, dextran was used as a coating for superparamagnetic iron oxide nanoparticles (SPIONs), employed in molecular imaging for early diagnosis of COPD. Functionalization with specific antibodies targeting CD86 and CD206 enables discrimination between pro-inflammatory and anti-inflammatory macrophages in LPS-induced murine models of COPD, offering high diagnostic sensitivity and excellent biocompatibility [52]. However, despite the numerous advantages offered by these systems, some studies report possible adverse effects related to the use of dextran, including anaphylactoid reactions [53], non-cardiogenic pulmonary edema, and acute renal failure [54]. Although rare, these events require careful toxicological evaluation prior to clinical translation. Overall, dextran proves to be a versatile and promising polymeric material for respiratory applications, capable of effectively integrating therapeutic function and diagnostic capability within a single nanostructured system. The main dextran-based formulations for COPD application are summarized in Table 3.

## 7. Epigenetics and miRNAs: Novel Targets and Delivery via Nanoparticles

Epigenetics is an emerging discipline for understanding the pathogenesis of COPD, offering new molecular targets for innovative therapeutic strategies. Epigenetic modifications, including DNA methylation, histone modification, and the expression of microRNAs (miRNAs) and small interfering RNAs (siRNAs), significantly influence gene expression without altering the DNA sequence. In COPD, these alterations contribute to the maintenance of chronic inflammation, oxidative stress, and cellular senescence, thereby worsening disease progression [55].

### 7.1. Role of miRNAs in COPD

miRNAs are small non-coding RNA molecules that negatively regulate gene expression at the post-transcriptional level. In COPD, various miRNAs have been identified as key regulators of inflammatory, fibrotic, and apoptotic processes, and mitochondrial dysfunction characteristic of the disease. For instance, miR-206 has been found to be overexpressed in dysfunctional skeletal muscle and plasma of COPD patients [56]. Sun Y. et al. [57] demonstrated that miR-206 was upregulated in lung tissues of COPD patients and smokers, suppressing the mRNA expression of Vascular Endothelial Growth Factor A (VEGFA) and Notch3, which are particularly important in cell fate determination and apoptosis [57]. miR-34a and miR-199a-5p levels were also significantly increased in the lungs of COPD patients. An increase in pulmonary endothelial and alveolar epithelial cells is observed, and cigarette smoke extract (CSE) induces endothelial cell apoptosis in a time- and dose-dependent manner. Kim et al. showed that miR-34a can bind to the 3′-UTR of the Notch1 gene, downregulating its expression in endothelial cells exposed to CSE. Furthermore, cadmium (Cd), present in cigarette smoke, exacerbates inflammation and pulmonary dysfunction, contributing to COPD progression by reducing the expression of miR-181a-2-3p and increasing inflammasome activity and inflammatory responses in lung cells [58]. The dysregulation of the insulin-like growth factor (IGF) system, which influences protein synthesis and ribosome number, may contribute to diseases such as cancer, muscle atrophy, and hypertrophy, which may also affect COPD patients. miRNAs play a key role in this process by regulating ribosomal function and ribosomal protein production. Specifically, in COPD patients, miR-424-5p inhibits protein synthesis and promotes muscle mass loss by inhibiting rRNA synthesis through the control of RNA polymerase I pre-initiation complex formation [59]. A recent study discovered the role of miR-146a, which is overexpressed in COPD patients and promotes the inflammatory response [60,61].

Another aspect that is worth highlighting is the impact of miRNAs in complications due to COPD treatment, such as ventilator-associated pneumonia (VAP) resulting from prolonged mechanical ventilation in the intensive care unit (ICU) [62]. In particular, an association has been identified between COPD, increased VAP risk, and ICU mortality, with Toll-like receptor 4 (TLR4) involved in the predisposition to VAP. Zhao et al. demonstrated that miR-1236 can bind to the 3′-untranslated region (3′-UTR) of TLR4 mRNA, increasing the risk of VAP in COPD patients [62]. The targeted modulation of miRNAs may thus represent a highly specific and potentially resolutive therapeutic strategy for COPD.

Multiple studies to date underscore the importance of miRNA regulation to counteract or prevent the effects of COPD. Two miRNAs, miR-320b and miR-150-5p, regulate essential cellular pathways in the development of COPD-associated lung cancer [63], showing a dual function: prevention of disease-associated tumorigenesis and a reduction in inflammation [64]. Similarly, miR-146a plays an anti-inflammatory role by repressing Toll-like receptor (TLR) and interleukin-1 (IL-1) signaling, inhibiting components such as TNF, IRAK1, and TRAF6, which in turn negatively regulate pro-inflammatory cytokines IL-8, IL-6, and IL-1β [61]. The use of nanoparticles (NPs) to deliver miR-146a has reduced IRAK1 and TRAF6 in human alveolar basal epithelial adenocarcinoma cells, showing an optimal therapeutic potential [65].

Another study, carried out in 2019 by Baker et al. [66], has shown that blocking miRNA-570-3p, whose levels increase due to oxidative stress, can restore sirtuin-1, a protein associated with cellular longevity. The inhibition of miR-570-3p by using analogs of microRNAs that function as silencing agents could help to counteract pulmonary and immune system aging, promoting normal cell proliferation over time and reducing pathological processes related to cellular senescence typical of COPD. It is known that COPD is characterized by chronic hypoxia, particularly mediated by hypoxia-inducible factor 1-alpha (HIF-1α). miR-186, also involved in tumor cell proliferation, reduces HIF-1α expression in pulmonary fibroblasts, promoting apoptosis of inflammatory fibroblasts [67]. In COPD, miR-27-3p acts on alveolar macrophages (AMs) influencing the release of pro-inflammatory cytokines, regulating TLR2/4 and inhibiting the pro-inflammatory nuclear receptor PPARγ [68]. Conversely, the reduced expression of miR-503 increases VEGF release by COPD-altered pulmonary fibroblasts, promoting vascular inflammation [69,70].

Another key aspect typical of COPD patients is pathological hyperplasia and tissue remodeling triggered by inflammation, which also leads to contractile dysfunction of skeletal muscle cells, worsening respiratory function. In vascular remodeling associated with COPD, miRNAs such as miR-197 regulate the fate of endothelial cells (ECs) and smooth muscle cells (SMCs). A reduction in miR-197 impairs the contractile markers of SMCs, influencing the contractile phenotype [71]. Shen et al. [72] demonstrated that miR-483-5p counteracts the cell proliferation induced by TGF-β, fibronectin, and α-SMA. These findings suggest that miR-483-5p may play a protective role in patients with COPD and could serve as a useful biomarker for early detection as well as a potential therapeutic target.

A final noteworthy aspect is the early identification of the disease to allow more effective treatment from its onset and, above all, to assess its prognosis to better tailor the therapeutic plan. Many studies show that several miRNAs represent potential biomarkers and therapeutic targets for COPD [73], as their dysregulation is associated with disease severity [74]. For example, miR-183-5p and miR-3177-3p are downregulated during disease progression [49], while miR-218-5p shows an inverse correlation with COPD severity and is reduced in smokers and affected patients [75]. Nanoparticle-based drug delivery systems, including miRNAs or antagomirs, can also be effectively utilized to target specific sites, thereby contributing to the efficient management of COPD [76].

### 7.2. Nanoparticles for the Delivery of Therapeutic Oligonucleotides

The clinical application of microRNAs (miRNAs) and interfering RNAs (siRNAs) requires effective and safe delivery systems capable of protecting these molecules from enzymatic degradation and ensuring their selective release into target cells. Nanoparticles have proven to be ideal tools for this purpose. In particular, lipid carriers, dendrimers, and biodegradable polymers have been used for the delivery of anti-inflammatory miRNAs and siRNAs aimed at reducing TNF-α, IL-6, and other pro-inflammatory mediators involved in COPD. It has been indicated that cationic polymeric nanoparticles can be successfully used for the delivery of miRNA-based drugs to negatively charged cell membranes in COPD and pulmonary diseases [34,60]. Pulmonary epithelial cells (A549), a model for COPD, were treated with a poly (glycerol adipate-co-ω-pentadecalactone)-based nanoparticle formulation encapsulating microRNA (miRNA) [77]. This cationic copolymer was designed to facilitate optimal interaction with the negatively charged cell membrane. The nanoparticles were prepared using a single-emulsion method followed by solvent evaporation, utilizing cationic precursors. The release efficacy of miRNA-146a was evaluated in vitro, showing efficient delivery of the genetic material to pulmonary cells. Furthermore, the miRNA retained its activity even after nanoparticle degradation, suggesting effective initial protection and functional release of the therapeutic cargo. Similarly, lipid carriers containing siRNA against IL-8 have demonstrated the ability to reduce neutrophilic infiltration in damaged lung tissues, offering sustained protective effects [78]. Another in vitro study employed CaP/PLGA nanoparticles as a targeted siRNA delivery system, revealing a promising strategy to treat pulmonary inflammation by protecting siRNA from degradation and reducing the expression of pro-inflammatory genes such as CCL-2, IP-10, and IFN-γ [79]. An emerging frontier involves the use of dextran-based nanogels as carriers for siRNA, with the aim of modulating gene expression of inflammatory mediators. The use of pulmonary surfactants, such as Curosurf, for surface decoration of these nanocarriers has been shown to improve penetration into alveolar macrophages and enhance intracellular delivery efficiency, while maintaining good cellular tolerability [80]. Khan et al. explored an innovative therapeutic strategy for COPD and asthma using PAMAM and PPI dendrimers modified with lipid substituents to encapsulate siRNA [81]. This combined formulation exerted a synergistic effect by selectively targeting the Tie-2 receptor in lung endothelial cells. Among the most effective formulations, those containing cholesterol C15 tails (in PAMAM) and C14–C15 chains (in PPE) showed high potency. In vivo tests confirmed the safety of the system, showing no increase in pro-inflammatory cytokines and no weight loss in treated mouse models.

These preclinical findings suggest that integrating epigenetic knowledge with nanotechnology may represent one of the most promising avenues for developing precision therapies in COPD, capable of acting upstream of inflammatory pathways and slowing disease progression more effectively than conventional approaches.

## 8. Physicochemical Properties of MOFs

Metal–organic frameworks (MOFs) represent a class of porous crystalline materials composed of metal ions or oxide clusters coordinated to multidentate organic linkers. Their hybrid organic–inorganic structure enables notable design versatility, allowing the modulation of porosity, chemical functionalization, and the achievement of high specific surface areas. These properties make MOFs highly promising candidates for applications in catalysis, chemical sensing, and controlled drug delivery [82,83,84,85].

MOFs consist of Secondary Building Units (SBUs) with variable geometries depending on the metal ion coordination (octahedral, prismatic, paddle-wheel, triangular) and organic linkers containing functional groups such as carboxylates, phosphonates, sulfonates, nitrogen-containing heterocycles, and amine and azo groups. The linkers may be ditopic, tritopic, tetratopic, or multitopic, reacting with metal centers endowed with multiple vacant or labile sites, leading to complex crystalline frameworks [86]. The final topology of the MOF depends on the interaction between the SBUs and the linkers, which can be optimized to obtain microporous (<2 nm) or mesoporous (2–50 nm) porosity. The rational selection of metals and linkers, along with control over the synthesis conditions (solvothermal, microwave, spray drying, continuous flow, solvent, temperature, pH, and modulators), allows precise tuning of size (from a few nanometers to micrometers), cavity distribution, and particle morphology [87]. The high surface-to-volume ratio promotes colloidal stability, biodistribution, and biocompatibility, while resistance to extreme pH conditions (up to 1.2 or 12.5) makes MOFs suitable for complex physiological systems [88,89]. The flexibility of the framework, induced by external stimuli (mechanical, thermal, light), enables dynamic behavior even in the absence of guests, a key property for responsive applications. MOFs, indeed, can be functionalized through covalent and non-covalent strategies to optimize interaction with the biological environment and develop multi-stimuli release (pH, heat, light), and the biodegradable nature of coordination bonds reduces the risk of long-term accumulation in tissues [90]. Drug loading and release efficiency depends on the type of interaction with the MOF matrix: weak interactions allow physical encapsulation, and medium interactions involve coordinated bonds, while strong interactions are obtained through post-synthetic modifications.

Recent advances have led to the creation of hybrid MOFs with functional nanoparticles, generating nano-MOFs with improved properties compared to conventional nanomedicines. These materials offer high loading capacity and biodegradability, and can be produced in crystalline or amorphous form, depending on application needs. The integration of biomolecules as ligands opens new perspectives for the design of bioinspired MOFs endowed with chirality, molecular recognition, self-assembly, and ion exchange or catalytic properties, capable of combining biological compatibility with advanced functionalities. The physicochemical properties of metal–organic frameworks (MOFs) require a more detailed discussion, particularly in the context of inhalation-based drug delivery for pulmonary diseases such as COPD. Key attributes, including particle size, shape, surface charge, porosity, and hydrophobicity, directly influence their aerodynamic behavior, determining how efficiently particles navigate the respiratory tract and deposit in specific lung regions. Additionally, interactions with the mucociliary clearance mechanisms are critical, as these dictate the residence time of the particles within the airways; for instance, highly mucoadhesive particles may enhance localized drug delivery but could also risk prolonged retention and toxicity. Finally, lung deposition efficiency, influenced by the mass median aerodynamic diameter (MMAD), is essential to ensure that therapeutic agents reach the lower airways or alveolar regions, where they can exert maximal therapeutic advantage. A comprehensive analysis of these parameters is necessary to assess the clinical applicability, safety, and effectiveness of MOFs and in pulmonary drug delivery systems.

### 8.1. pH-Sensitive Release and Alveolar Targeting

One of the most innovative features of MOFs lies in the possibility of designing drug delivery systems sensitive to specific environmental stimuli, such as pH. In the inflammatory microenvironment typical of COPD, a reduction in tissue pH is observed, particularly at the alveolar level, due to the accumulation of inflammatory cells and the production of lactic acid. pH-sensitive MOFs are designed to remain stable at physiological pH; instead, they degrade in acidic environments (pH 6.5 or lower), thereby releasing the drug selectively. This strategy allows for maximizing therapeutic efficacy and minimizing systemic exposure, significantly improving the safety profile of administered drugs. Alveolar targeting represents a key strategy to improve the biodistribution of MOFs within the lungs. This can be achieved through surface functionalization of MOFs with specific ligands, such as peptides, antibodies, or aptamers, capable of recognizing overexpressed markers in inflamed epithelial cells or alveolar macrophages. Additionally, the size and surface charge of the particles play a determining role in alveolar deposition and penetration of mucosal barriers.

Inhalable formulations based on MOFs, combined with active targeting strategies and pH-sensitive release, are opening new possibilities for selective and highly effective treatments of COPD, minimizing interactions with healthy tissues and optimizing the therapeutic response in pathological regions.

### 8.2. MOFs in COPD: Structure, Mechanisms, and Applications

Metal–organic frameworks (MOFs) represent one of the most promising frontiers of nanomedicine for chronic respiratory diseases such as COPD. They are hybrid materials composed of metal ions or clusters coordinated with organic ligands to form highly porous and crystalline structures. Their physicochemical properties, including high surface area, tunable porosity, and the possibility of post-synthetic functionalization, make them particularly suitable for targeted drug delivery, transport of sensitive molecules, and the development of smart theranostic systems.

The interplay between COPD and MOFs lies primarily in the development of MOF-based systems for targeted therapy, diagnosis, and biomolecule stabilization in the inflamed pulmonary environment. Recent advances suggest that MOFs can mitigate oxidative stress by scavenging reactive oxygen species (ROS) or delivering antioxidants directly to inflamed tissues. Moreover, their ability to carry nucleic acid-based therapeutics, such as siRNA or miRNA, presents a novel strategy to modulate gene expression pathways implicated in COPD pathogenesis. MOFs are increasingly attracting interest in medicine, particularly for oral and intravenous drug administration, but their inhalation-based application remains underexplored [91]. However, spray drying represents a promising technology for producing respirable microparticles from nano-MOFs, transforming them into hollow, spherical superstructures suitable for pulmonary delivery [92]. Various sources in the literature discuss the optimization of spray drying, including a comprehensive review by Troyano J. et al. [93]. This continuous and scalable technique allows the production of dry powders for inhalers without the use of solvents or complex surfactants, reducing costs and time. MOFs produced in this way can disintegrate in the lungs, release the drug in acidic environments (such as macrophages or tumors), and effectively reach cells via endocytosis. Previous studies have demonstrated the versatility of this technique with several types of MOFs, such as UiO-66, MIL-88, MOF-74, ZIF-8, and others.

In summary, the combination of MOFs and spray drying opens new perspectives for targeted therapeutic inhalation, enabling controlled and site-specific drug release directly into the lungs.

### 8.3. Toxicity, Biocompatibility, and Regulatory Challenges

Metal–organic frameworks (MOFs), thanks to their porous structure, modulable composition, and targeted functionalization, represent a promising class of materials for therapeutic applications, particularly for inhalation delivery. However, their systemic biocompatibility remains a research priority, as once inhaled, MOFs and engineered nanomaterials in general can cross the pulmonary epithelium and reach the bloodstream, with potential accumulation in distal organs, interference with the vascular endothelium, and even neurotoxic effects through translocation along the olfactory nerves [94,95,96].

The cytotoxicity of MOFs depends on key factors such as the metal used and the chemical nature of the ligands. Some studies have shown that the release of metal ions or organic components can trigger undesired inflammatory responses and oxidative stress [97,98]. In particular, amine ligands prove to be more reactive and potentially toxic compared to carboxylic ligands, which are generally more stable and biocompatible [99]. Some bio-MOFs incorporate biologically active ligands that participate in physiological cycles or exert antimicrobial actions, while maintaining controlled toxicity. Biocompatible coatings and surface modifications are essential to reduce systemic toxicity and increase in vivo tolerability [100], although the supramolecular architecture of MOFs, which allows for fine design, requires strict synthesis conditions. Unlike conventional inorganic nanoparticles, often subject to uncontrolled release of toxic ions and colloidal instability, MOFs offer greater stability in solution and more predictable release kinetics. Moreover, the use of essential metals such as iron, zinc, and magnesium improves the safety profile, while elements such as zirconium and titanium, although poorly absorbable, exhibit low acute toxicity (LD_50_ > 25 g/kg), making them suitable even for dermocosmetic applications [101]. A recent 2024 study conducted by Chen et al. highlighted how photo-aging processes can significantly modify the physicochemical and biological properties of metal–organic framework (MOF)-based nano-systems, in particular nano-ZIF-8 (nZIF-8), enhancing their biocompatibility [102]. In the study, it was observed that prolonged exposure to simulated solar radiation induces a marked reduction in particle size (from ~200 to ~100 nm), accompanied by substantial structural disintegration. These morphological and surface transformations resulted in a reduction in oxidative cytotoxicity in human pulmonary epithelial cells BEAS-2B, attributable to a lower intracellular production of reactive oxygen species (ROS). Therefore, gene expression analysis revealed a decrease in the inflammatory and cytotoxic responses induced by aged nZIF-8, demonstrating its ability to negatively modulate genes associated with the pathogenesis of COPD, including pro-inflammatory cytokines and specific markers such as SERPINA1, HBEGF, and DIO2 [103]. These data suggest that environmental aging processes may attenuate the potentially adverse biological impact of these materials, making them more biocompatible and safe for long-term biomedical and environmental applications. Overall, the results highlight the importance of considering the effects of environmental aging in the safety assessment of nanostructured MOFs, not only for therapeutic use but also for industrial and environmental applications, where prolonged exposure to sunlight or other environmental factors can significantly modify their toxicological profile. However, it should be considered that systemic toxicological evaluation of MOFs includes studies on biodistribution, tissue accumulation, excretion, and degradation, which are essential to define the risk–benefit balance in clinical applications. Biodegradability is a crucial aspect: MOFs must be able to break down into nontoxic products after fulfilling their therapeutic function. It has been observed that some formulations are rapidly cleared by alveolar macrophages, while others persist longer in pulmonary tissues, raising concerns about their long-term safety. Surface modifications, such as PEGylation or the use of lung-targeting ligands, can further enhance biocompatibility and reduce immunogenicity. Moreover, MOFs can be engineered to degrade into nontoxic byproducts under physiological or pathological conditions, reducing the risk of long-term accumulation in lung tissues. It is therefore necessary to develop specific guidelines for hybrid nanomaterials, including toxicokinetic tests, immunogenicity, controlled release, and interaction with the biological environment. Interdisciplinary collaborations among academia, industry, and regulatory agencies are essential to build a clear and safe regulatory framework. Therefore, a thorough evaluation of both acute and chronic toxicity profiles, as well as dose-dependent effects, is essential to ensure the safe translation of these systems into clinical use for conditions such as COPD.

In summary, although MOFs represent a promising therapeutic innovation, their success will depend on the ability to ensure safety, reproducibility, and compliance with regulatory requirements—indispensable elements for their future clinical adoption.

### 8.4. Relevant Preclinical Studies on MOFs in COPD Treatment

Numerous preclinical studies have evaluated the efficacy of MOFs as therapeutic platforms in COPD, with promising results both in vitro and in vivo. Among the most investigated formulations are Fe-MIL-100, UiO-66, and nanoMIL-89, each with distinct physicochemical characteristics and pharmacokinetic profiles. Strzempek et al. [104] developed the MOF Fe-MIL-100 for inhalable delivery of theophylline, a drug belonging to the methylxanthine class used as a bronchodilator in the treatment of chronic respiratory diseases such as asthma, chronic bronchitis, and COPD. The material was synthetized through the reaction between trimesic acid and ferrous chloride, followed by thermal activation. The drug was encapsulated by exposing the MOF to an acidic solution, obtaining the compound TP@Fe-MIL-100. XRD analysis confirmed the preservation of the crystalline structure, with a decrease in peaks, indicative of drug incorporation. Under simulated pulmonary conditions, drug release was controlled (46% in 8 h), whereas free theophylline showed explosive release (95% in 3 h). Cytotoxicity tests demonstrated good biocompatibility, with only a moderate reduction in cell viability at high concentrations. Fernandez-Paz et al. [91] used MIL-100 as a pulmonary delivery system (Ma-MIL-100 MS mannitol microspheres) based on microspheres obtained by spray drying. They tested three biocompatible carbohydrates (dextran, α-cyclodextrin, and mannitol) with negative surface charge. Mannitol proved to be the most suitable thanks to its excellent spray-drying properties and regulatory approval. The final formulation showed colloidal stability and appropriate particle size for deep lung deposition. In vivo studies in rats showed that the nano-MOF effectively distributed in the respiratory tract, accumulating in bronchioles and alveoli. Using isoniazid, an antibacterial drug mainly used for the treatment and prevention of tuberculosis, the system demonstrated improved pulmonary drug distribution and potential for reducing the development of drug resistance.

CD-MOFs also have attracted much attention in drug delivery because they incorporate the porous features of MOFs and the encapsulation capability of CD for drug molecules. The metal ions in CD-MOFs may bring additional therapeutic effects regarding CD hydrogel [105]. For instance, γ-cyclodextrin-based MOFs (CD-MOFs) loaded with budesonide (BUD) were modified with cholesterol to improve aerodynamic properties for dry powder inhalers. In vivo animal experiments demonstrated that these CHO-CD-MOF-BUD formulations were well-tolerated and effectively delivered the drug to the lungs, suggesting their potential as inhalable carriers for COPD treatment. Jarai et al. [106] proposed a drug delivery system based on UiO-66, a metal–organic framework (MOF) composed of zirconium and terephthalic acid that was post-synthetically loaded with Rhodamine B or dexamethasone, a synthetic corticosteroid with anti-inflammatory, immunosuppressive, and antiallergic effects, used in the treatment of numerous diseases, including chronic inflammatory diseases, and, among others, to prevent or reduce pulmonary inflammation in COPD exacerbations. The UiO-66 NPs exhibit nanometric size and negative surface charge, which promote phagocytosis and support alveolar clearance. Despite containing structural defects, they show high loading efficiency. Drug release was faster in acidic environments, indicating a pH-dependent release mechanism, which is useful in pathological conditions such as tumors. Furthermore, in the same study, UiO-66 nanoparticles were tested on pulmonary epithelial cells and alveolar macrophages, revealing excellent in vitro biocompatibility even at high concentrations. The NPs were readily internalized by macrophages through active processes and showed intracellular release of the fluorescent cargo, suggesting effective degradation in the cytoplasm. Moreover, the absence of pro-inflammatory cytokine secretion (TNF-α and IL-6) indicates a minimal immune response. These results highlight the suitability of UiO-66 NPs as a safe and biocompatible platform for inhalable drug delivery. A previous study by Avci-Camur et al. optimized the synthesis of UiO-66 in a mixture of water and acetic acid, contributing to solving one of the main obstacles to the industrial-scale production of nano-MOFs: the absence of efficient water-based synthetic methods [107]. Mohamed et al. developed nanoMIL-89 for targeted delivery of sildenafil, a vasodilator belonging to the phosphodiesterase type 5 (PDE5) inhibitor class, used in the treatment of pulmonary arterial hypertension (PAH), which is one of the most frequent complications in COPD patients [108]. Unlike free sildenafil, the drug release in human plasma was prolonged up to 96 h. Biocompatibility and preservation of pharmacological activity were confirmed through in vitro testing. Another example of MOF application in COPD is the use of ZIF-8 (Zinc Imidazolate Framework-8) in a study conducted by Zhou et al., which developed the CeO_2_@ZIF-8/Au system, also referred to as CZA, representing an innovative approach due to its multifunctional and tunable activity in the treatment of chronic inflammatory diseases. This nanocomposite consists of gold nanoparticles (Au) with peroxidase-like activity, and cerium dioxide (CeO_2_) encapsulated within a ZIF-8 MOF matrix, and presents controlled release of CeO_2_ in acidic environments, such as the inflamed ones typical of COPD [109]. Moreover, gene expression analysis showed that the treatment with CZA downregulated mRNA levels of caspase-3, suggesting protection against oxidative stress-induced apoptosis. At the same time, a significant reduction in inflammatory cytokines such as IL-6 and TNF-α was observed in LPS-treated cell models, highlighting the ability of CZA to attenuate the inflammatory response. In summary, the CeO_2_@ZIF-8/Au (CZA) system offers a promising approach for the treatment of COPD, thanks to its ability to dynamically modulate the oxidative–inflammatory microenvironment, promote tissue regeneration, and improve therapeutic safety through controlled release of the active drug. These characteristics make it an ideal candidate for future developments in respiratory nanomedicine. Two novel mixed-ligand Co(II) coordination polymers were investigated for their therapeutic activity in COPD. These MOFs modulated the AMP-activated protein kinase (AMPK) signaling pathway, which plays a crucial role in cellular energy regulation and inflammation. By influencing this pathway, the MOFs exhibited anti-inflammatory effects, highlighting their potential in managing COPD-related inflammation. Furthermore, treatment with the compound significantly reduced the accumulation of ROS in alveolar epithelial cells [110]. MOFs have also been used for diagnostic purposes for COPD. An engineered bimetallic MOF nanomaterial was employed as a matrix in laser desorption/ionization mass spectrometry to extract serum metabolic fingerprints from COPD patients. This approach enabled the identification of potential biomarkers and facilitated the differentiation between COPD patients and healthy controls, demonstrating the diagnostic capabilities of MOF-based systems [111].

These preclinical results suggest that MOFs could constitute a new class of carriers for targeted drug delivery in COPD. However, their clinical translation requires further evaluation of safety, biodegradability, and immune response in human models.

### 8.5. Theranostics: Integrated Diagnosis and Therapy

Theranostics represents one of the most innovative applications of MOFs in respiratory medicine (especially for asthma and COPD), oncology, and infectious and viral diseases, combining both therapeutic and diagnostic functions into a single platform [112]. This dual capacity is particularly useful in COPD, where real-time monitoring of drug distribution and therapeutic response can guide more personalized and timely interventions. MOFs can be loaded with drugs and simultaneously integrated with imaging agents such as fluorescent probes, radionuclides, or magnetic nanoparticles. This multifunctionality enables visualization of the nanocarrier’s biodistribution, assessment of its effectiveness, and adjustment of the therapy based on the patient’s response.

MOFs functionalized with metals could be useful for magnetic resonance imaging (MRI), providing a detailed diagnostic signal while simultaneously releasing the targeted anti-inflammatory molecules. Although the use of MRI as a diagnostic tool in chronic lung diseases such as COPD is still under investigation and thus not yet widely adopted in clinical practice [113], it is becoming increasingly useful in complex cases thanks to recent technological advances. MRI allows detailed imaging of lung structure without ionizing radiation, evaluation of blood perfusion, and analysis of associated cardiovascular alterations such as pulmonary hypertension. These applications make it a promising tool for follow-up and advanced disease management. The MOF Fe-MIL-101-NH_2_, as demonstrated by Wyszogrodzka et al., enables the prolonged release of isoniazid (an antibiotic used in TB treatment) directly into the cellular cytoplasm, overcoming the rapid dissolution of the free drug, and also offers diagnostic potential as a contrast agent for imaging. This nanomaterial has also been successfully used as a delivery system for theophylline, a drug used for asthma and COPD [114]. Rieter et al. [115] highlighted the potential of MOFs at the nanoscale as multimodal imaging probes, synthesizing nanorods and nanoplates through microemulsion of GdCl_3_ and bis(methylammonium)-benzene-1,4-dicarboxylate in the presence of a cationic medium (CTAB/isooctane/1-hexanol/water). They proposed that a high metal load in NMOFs is advantageous as a contrast agent for MRI, with MOF nanorods showing water signal efficiency superior to the clinical product OmniScan [116]. A study conducted by Hairu Lin et al. [111] described the use of MOFs as a matrix for laser desorption/ionization (LDI), which, through the metabolomic analysis of biological fluids, represents a particularly relevant diagnostic system for COPD. Upon irradiation with a focused laser pulse, the MOF absorbs the energy and transfers it to the adsorbed analytes, facilitating their desorption and simultaneous ionization in a gentle manner that preserves molecular integrity. The resulting ions are then separated by the mass spectrometer based on their mass-to-charge ratio (*m*/*z*), yielding a precise and sensitive metabolic fingerprint. Thanks to the chemical versatility of MOFs, this method allows improved yield, selectivity, and reproducibility in biomarker identification, opening new perspectives for non-invasive molecular diagnostics.

In particular, the study developed a metal oxide-based nanomaterial, derived from a MOF (CoFeNMOF-D), which was used as a matrix for LDI-MS to profile the serum metabolome of patients with COPD and healthy controls (HCs). The analysis of metabolic fingerprints, integrated with machine learning algorithms, enabled a clear separation between the two groups. Four metabolites, quinolinic acid, glyceric acid, taurine, and pyroglutamic acid, were identified as significantly downregulated in COPD and selected as potential diagnostic biomarkers [111]. Diagnostic models based on these markers achieved area under the curve (AUC) values of 0.931 in the training set and 0.978 in the validation set. Furthermore, pathway analysis and pathogenic mechanisms related to these biomarkers supported the use of LDI-MS-based molecular diagnostics for the clinical diagnosis of COPD. In conclusion, it is important to emphasize that the unique capacity of theranostic MOFs to undergo selective activation in response to specific stimuli, such as acidic pH, reactive oxygen species (ROS), or inflammatory enzymes, places them as valuable tools for the early detection of exacerbations and the dynamic evaluation of therapeutic efficacy in COPD. Although the clinical application of theranostics in COPD patients is still in its early stages, preclinical data suggest significant potential for optimizing patient management and outcomes.

## 9. Future Perspectives and Conclusions

Research on nanomaterials for the treatment of COPD is currently in an intense phase of innovation and development. The preclinical results obtained with nanoparticles of various types, ranging from biodegradable polymers to dendrimers, from liposomes to theranostic systems, demonstrate how nanostructural engineering can offer advanced solutions to therapeutic problems that have remained unresolved until now. In this context, metal–organic frameworks stand out for their structural versatility, high loading capacity, pH-sensitive release, and possibilities for active targeting and integrated diagnosis. The most promising future perspectives include the optimization of inhalable formulations, the development of multifunctional MOFs for theranostic applications, and integration with artificial intelligence tools for the rational design of nanocarriers. Moreover, the use of MOFs for the delivery of therapeutic RNAs and epigenetic modulation opens new avenues in precision medicine, enabling intervention in specific molecular pathways involved in COPD progression. However, the clinical translation of these technologies will require coordinated efforts on multiple fronts: the standardization of synthesis methodologies, rigorous evaluation of long-term safety, and the development of an ad hoc regulatory framework. It will also be essential to involve patients in the decision-making processes, promoting well-designed clinical trials that evaluate not only therapeutic efficacy but also quality of life and treatment acceptability.

In conclusion, nanomaterials, and in particular MOFs, represent one of the most advanced and promising frontiers for the active release of drugs for the treatment of COPD. Their ability to combine targeted release, spatiotemporal control, and diagnostic functionality could revolutionize the treatment of this complex and multifactorial disease, bringing respiratory medicine closer to an increasingly personalized, effective, and sustainable approach.

## Figures and Tables

**Table 1:** Main PLGA-based nanoparticle formulations for COPD applications.

Formulation	Loaded Molecules	Function	Application in COPD	Reference
HA@PLGA NPS	Polymyxin B	Antibiotic anti-Gram-negative	Improved mucus penetration and deep lung delivery	[13]
PLGA/poly(vinyl-sulfonate-co-vynyl-alcohol) NPs	Salbutamol	β2-agonist bronchodilator	Controlled release and enhanced pulmonary retention	[14]
Lipid–polymer hybrid Nps	Mn-porphyrin dimer (MnPD)	Antioxidant + gene delivery	Increased HDAC2 levels	[15]
PLGA-PEG immunoconjugated NPS	Ibuprofen	Anti-inflammatory	Reduced neutrophilic inflammation in cigarette smoke model	[16]
PLGA-PVA NPs	Thymoquinone	Anti-inflammatory, antifibrotic	Protected against bleomycin induced fibrosis	[17]
PLGA lipid–polymer microparticles	Oridonin	Anti-inflammatory,	Reduced inflammation in COPD and asthma	[18]
NCMPs with PLA NPs, coated with chitosan	Curcumin	Antioxidant	Reduced TNFα response and extended pulmonary retention	[20]

**Table 2:** Liposomal and nanoemulsion-based formulations for COPD applications.

Formulation	Loaded Molecule	Function	Application in COPD	Reference
Liposomal	Budesonide	Anti-inflammatory	Improved lung accumulation and reduced bronchial inflammation	[23]
Liposomal	Budesonide	Corticosteroid release	Enhanced compliance	[24]
Liposomal	Salbutamol	Bronchodilation	Enhanced retention time and site-specific delivery	[25]
Liposomal	DPPC	Passive lung targeting	Prolonged lung retention and absorption	[26,27]
Liposomal	N-acetylcysteine (NAC), glutathione, α-tocopherol, SOD, CAT	Anti-inflammatory, antifibrotic	Improved enzymatic activity and reduced oxidative lung injury	[30]
Liposomal	Curcumin + hyaluronan/chitosan	Oxidative stress protection	Enhanced cell proliferation and antioxidant protection	[31,32]
Liposomal	Pegylated liposomes	Targeted delivery via surface modification	Reduced macrophage uptake and enhanced systemic circulation	[33]
Nanoemulsion	Agarwood essential oil	Anti-inflammatory	Reduced pro-inflammatory cytokines and activated protective pathways	[34]
Nanoemulsion	Budesonide	Enhanced pulmonary delivery of corticosteroids	Better lung deposition	[35]

**Table 3:** Main dextran-based formulations for COPD application.

Formulation	Loaded Molecules	Function	Application in COPD	Reference
Dextran–budesonide conjugates (ester-linked)	Budesonide	Prolonged release via enzymatic cleavage by carboxylesterase 1	Improved therapeutic efficacy and patient compliance	[47]
Crosslinked dextran NPs	Anti-inflammatory or antioxidant agent	pH-responsive and prolonged retention in airways	Targeted drug delivery in inflamed pulmonary tissue	[48]
Acetylated dextran microparticles	Budesonide, curcumin	Acid-sensitive release in lysosomal compartments	Ros scavenging and macrophage polarization (M2)	[49,50]
Dextran-based nanogels	Dexamethasone	Swelling and muco-penetrating ability	ICAM-1-targeted therapy for lung inflammation	[51]
Dextran-coated SPIONS	Diagnostic agents + CD86/CD206 antibodies	Molecular imaging; discrimination between macrophage subtypes	Theranostic tool for early COPD diagnosis	[52]

## Data Availability

Not applicable.
